# Urinary and Double Incontinence in Cognitively Impaired Patients: Impacts on Those Affected and Their Professional Caregivers

**DOI:** 10.3390/jcm12103352

**Published:** 2023-05-09

**Authors:** Anke Kirsten Jaekel, Theresa Maria Rings, Franziska Schmitz, Franziska Knappe, Alix Tschirhart, Franziska Isabelle Winterhagen, Ruth Klara Maria Kirschner-Hermanns, Stephanie C. Knüpfer

**Affiliations:** 1 Department for Neuro-Urology, Clinic for Urology, University Hospital Bonn, 53127 Bonn, Germany; 2 Neuro-Urology, Johanniter Neurological Rehabilitation Center ‘Godeshoehe e.V.’, 53177 Bonn, Germany

**Keywords:** urinary incontinence, double incontinence, caregiver, nursing home, cognitive impairment, questionnaire

## Abstract

Urinary or double incontinence in frail elderly people is common and leads to a reduction in quality of life and an increased burden on the patients’ caregivers. Up to now, no special instrument has been available to assess the impact of incontinence on cognitively impaired patients and their professional caregivers. Thus, the outcomes of incontinence-specific medical and nursing interventions for cognitively impaired individuals are not measurable. Our aim was to investigate the impacts of urinary and double incontinence on both the affected patients and their caregivers using the newly developed “International Consultation on Incontinence Questionnaire Cognitively Impaired Elderly” (ICIQ-Cog) tool. The severity of incontinence was measured by incontinence episodes per night/per 24 h, the type of incontinence, the type of incontinence devices used, and the proportion of incontinence care out of total care; all these measures were correlated to the ICIQ-Cog. Incontinence episodes per night and the proportion of incontinence care out of total care showed significant correlations with the patient- and caregiver-related ICIQ-Cog scores. Both items have negative effects on patient quality of life and caregiver burden. Improving nocturnal incontinence and reducing the need for incontinence care overall can decrease the incontinence-specific bother of affected patients and their professional caregivers. The ICIQ-Cog can be used to verify the impacts of medical and nursing interventions.

## 1. Introduction

Incontinence is a prevalent geriatric syndrome [1,2]. Urinary incontinence (UI) affects up to 80% of nursing home residents [1,2,3]. About 65% of elderly nursing home residents suffer from double incontinence, defined as concurrent UI and fecal incontinence [2]. The causes of urinary incontinence are multifactorial and not only age-related. Factors beyond the genitourinary tract, such as pre-existing illnesses, medication, neurological conditions, and a loss of functional abilities, can cause urinary incontinence [4]. The risk factors of fecal incontinence are age, loss of functional abilities, reduced mobility, fecal consistency, dysfunction of the bowel, dementia, depression, neurological conditions, diabetes mellitus, and chronic disease [4].

Incontinence has a significant impact on the affected persons, as it is associated with complications such as pressure ulcers [2], falls [5], and urinary tract infections [6,7]. It is also associated with a higher risk of frailty [8], and increased mortality has been demonstrated in several studies [1,9]. Cognitively impaired and elderly persons suffer more often from incontinence [3,10,11], and incontinence leads to a decrease in the quality of life of the affected person [3]. As more and more persons with cognitive impairment are cared for in nursing homes in our aging society, incontinence in this patient cohort represents a problem of increasing importance for those affected and for their caregivers. Incontinence results in an increased burden placed on caregivers [12], combined with increasing challenges related to care and financial issues [13]. Therefore, knowledge of the specific impacts of incontinence and a tool that can monitor the success of medical interventions in this patient cohort are both necessary in order to make therapeutic interventions more efficient.

Established questionnaires such as the IIQ-7 [14] and the ICI-Q (http://iciq.net/, accessed on 23 January 2023) are available to assess incontinence-related quality of life, but their instruments do not cover the specific life situation of cognitively impaired nursing home residents. Furthermore, members of this patient cohort are often not able to complete the questionnaires. Many studies exist on informal caregiver burden [12,15,16]. These studies have used the Zarit Caregivers Burden Scale, but this was developed to assess caregiver burden among family members. We did not find an instrument that could be used to assess professional caregivers’ incontinence-related burden. Therefore, the impact of incontinence on professional caregivers has not been assessed using specially adapted questionnaires.

Before the development of the International Consultation on Incontinence Questionnaire Cognitively Impaired Elderly (ICIQ-Cog) tool [17], no instruments existed to measure the incontinence-related quality-of-life impairment of cognitively impaired elderly persons.

Our aim was to investigate the impact of urinary and double incontinence on the affected persons, and the incontinence-related burden on their caregivers, using the newly developed ICIQ-Cog.

## 2. Materials and Methods

### 2.1. Patients and Assessment

We included 60 patients from 2 nursing homes in our analysis, based on an open multicenter observational study within the development and validation process of the ICIQ-Cog [17]. The inclusion criteria were the presence of incontinence, cognitive impairment, and written informed consent from the respective legal representatives. The exclusion criteria were palliative treatment and an indwelling catheter. A test administrator interviewed the participants, explained the study, and requested consent for participation. The test administrator assessed the cognitive impairment of the participants using the Mini-Mental State Examination (MMSE) [18] supplemented with the Global Deterioration Scale (GDS) [19]. We chose a threshold of 27 for the MMSE so as to achieve an appropriate balance between sensitivity and specificity. The threshold of GDS, which assesses the severity of the cognitive impairment, was set at 3. Data on concomitant diagnoses, length of nursing home stay, and duration of incontinence were obtained from the participants’ records. The demographic data and work experience of the caregivers were recorded to derive the characteristics of the rater.

The caregivers filled in the new ICIQ-Cog assessment tool [17]. The ICIQ-Cog questionnaire consists of three parts. A general part, containing 7 items, records the severity of incontinence and the type and proportion of incontinence care. A patient-directed part (ICIQ-Cog-P) is included, with 12 questions on the incontinence-specific burden of the patient as a third-party assessment, and a care-related part (ICIQ-Cog-C) with 4 questions on the care effort. ICIQ-Cog-P and ICIQ-Cog-C use 4-point Likert scales. High point scores indicate a high burden. The maximum score of the ICIQ-Cog-P is 48 and that of the ICIQ-COG-C is 16.

Derived from our aim, the main hypothesis was the following assumption: the more severe the incontinence, the greater the burden on patients and caregivers.

To verify our hypotheses, we measured the severity of incontinence using the following parameters: type of incontinence (urinary or double incontinence), types of incontinence devices used, frequency of incontinence episodes per night, frequency of incontinence episodes per 24 h, and proportion of incontinence care out of total care. The incontinence-related quality-of-life impairment of the patients was measured using the ICIQ-Cog-P, and the incontinence-specific burden on their caregivers was measured using the ICIQ-Cog-C.

The primary endpoint was the correlation between the number of nocturnal incontinence episodes and the sum score of the ICIQ-Cog-P and -C. The secondary outcomes were the correlations between the type of incontinence (urinary or double incontinence), types of incontinence devices used, frequency of incontinence episodes per 24 h, and the proportion of incontinence care out of total care and ICIQ-Cog-P and -C, i.e., the incontinence-related quality-of-life impairment of the patient and the incontinence-specific burden on their caregivers, respectively. An expense allowance of EUR 10 was paid to the caregivers. The ethics committee of the university clinic of Bonn, Germany, issued ethical approval for this study (EK 182/13).

### 2.2. Statistical Analysis

The descriptive analysis was performed using SPSS^®^, version 29.0 (IBM Corp., Armonk, NY, USA). All other analyses were performed using the R statistical programming language [20]. We checked the normality assumption for the studentized residuals from the linear models using quantile–quantile plots. No relevant deviations could be identified. The correlations between the incontinence episodes per night/per 24 h and the proportion of incontinence care out of the total care were investigated with the ICIQ-Cog using Pearson’s correlation coefficient, while linear regression models were used to estimate the impact. To compare the impacts of the type of incontinence, the incontinence devices used, and the binary calculation of incontinence episodes per night, the Wilcoxon rank-sum test and the independent-sample *t*-test were used. The post hoc power analysis for the primary outcome (correlation between the number of nocturnal incontinence episodes and the sum score of the ICIQ-Cog-P and -C) revealed acceptable power of 0.88 and 0.76, respectively (significance level 0.05, *n* = 16, and correlation coefficients of 0.69 and 0.62, respectively). A *p*-value < 0.05 was considered statistically significant.

## 3. Results

### 3.1. Patients’ Characteristics and Results of ICIQ-Cog-P and -C

Our patient cohort from two nursing homes consisted of 83% women (50/60) and 17% men (10/60). Out of the caregivers, 98.3% (59/60) were female and 1.7% (1/60) were male. The characteristics of both groups are shown in detail in Table 1.

**Figure 1:**
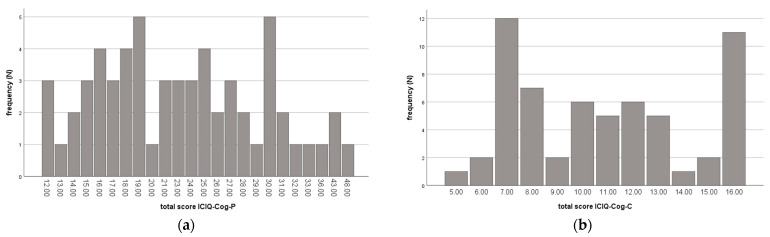
Frequency distributions of the ICIQ-Cog-P (**a**) and -C (**b**).

The results of the summed Likert scale scores for the ICIQ-Cog-P and ICIQ-Cog-C are shown in Table 2. The frequency distribution is summarized in Figure 1.

### 3.2. Correlations and Effects of Severity of Incontinence and the ICIQ-Cog

#### 3.2.1. Type of Incontinence (Urinary or Double Incontinence)

In our patient cohort, 41.7% (25/60) had urinary incontinence, and 58.3% (35/60) had double incontinence. The mean ICIQ-Cog-P sum score of patients with urinary incontinence was 23.5 (SD 7.33). The mean ICIQ-Cog-P sum score of patients with double incontinence was 23.0 (SD 8.3). There was nearly no difference in the sum score of the ICIQ-Cog-P between the types of incontinence, and the *t*-test showed no significant difference between the groups (Table 3). The mean ICIQ-Cog-C sum score of patients with urinary incontinence was 9.84 (SD 3.2) and that of those with double incontinence was 11.46 (SD 3.48). The difference in these ICIQ-Cog-C sum scores was not significant (Table 3). We could not show a significant correlation between the type of incontinence and the results of the ICIQ-Cog-P and -C within our cohort.

#### 3.2.2. Types of Incontinence Devices Used

The frequency distributions of the incontinence devices used were 8.3% (5/60) pants (all-around), 25.0% (15/60) small incontinence pads, 63.3% (38/60) large incontinence pads, and 3.3% (2/60) others. In total, 80% (4/5) of those wearing pants, 61% (23/38) of those using large pads, and 46% (7/15) of those using small pads had double incontinence. The mean ICIQ-Cog-P sum score of patients using pants was 20.6 (SD 5.94) and that of those using other incontinence devices was 23.45 (SD 8.0). The *t*-test showed no significant differences between the incontinence device groups (Table 3). The mean ICIQ-Cog-C sum score of patients using pants was 10.6 (SD 1.67) and that of those using other incontinence devices was 10.8 (SD 3.56). The mean ICIQ-Cog-C sum score was nearly the same between the groups (Table 3). There was no significant difference between groups of incontinence device users in terms of the results for the ICIQ-Cog-P and -C.

#### 3.2.3. Frequency of Incontinence Episodes per Night and per 24 h

For the assessment of nocturnal incontinence episodes, 26.7% (16/60) valid observations were available. The high number of missing values is because the ICIQ-Cog links the question about nocturnal episodes to the completion of a bladder diary (BD), but BDs were not completed in the facilities in question. The frequency distribution of nocturnal episodes was as follows: no nocturnal incontinence 3.3% (2/60), one episode 3.3% (2/60), two episodes 11.7% (7/60), three episodes 1.7% (1/60), and four episodes 6.7% (4/60).

The nocturnal incontinence episodes correlated strongly and positively with the summed scores of the ICIQ-Cog-P and -C, with Pearson correlation coefficients of r = 0.69 and r = 0.62. The correlations were significant. Thus, the summed score of the ICIQ-Cog-P changed by 4.78 points when one incontinence episode per night was added. An additional incontinence episode per night caused an increase of 1.22 points in the ICIQ-Cog-C (Table 4).

In the binary analysis (presence of nocturnal incontinence; yes/no), there was a significant difference between the groups regarding the sum score of ICIQ-Cog-C. Patients with nocturnal urinary incontinence had significantly higher ICIQ-Cog-C scores than patients without nocturnal urinary incontinence. We cannot demonstrate this binary relationship in the ICIQ-Cog-P using our data. For the assessment of incontinence episodes per 24 h, 40.0% (24/60) valid observations were available. This was also linked to the presence of a BD in our questionnaire, and so only a few data were available. The frequency distribution was as follows: one incontinence episode per 24 h 1.7% (1/60), two episodes 5.0% (3/60), three episodes 3.3% (2/60), and four or more episodes 30.0% (18/60). For incontinence episodes per 24 h, we did not find clear positive or negative correlations with the scores for ICIQ-Cog-P (r = −0.07, CI −0.46; 0.34) and ICIQ-Cog-C (r = 0.16, CI −0.26; 0.53). Therefore, the impact of the number of incontinence episodes per 24 h on the ICIQ-Cog-P and -C was not assessable using our data (Table 4).

#### 3.2.4. Proportion of Incontinence Care Out of Total Care

The impact of the proportion of incontinence care out of total care on the ICIQ-Cog showed positive correlations for the ICIQ-Cog-P and the ICIQ-Cog-C. The correlation coefficient was r = 0.27 for the former and r = 0.46 for the latter (Table 4). The linear regression model confirmed this correlation. If the proportion of incontinence care increased by 10%, the ICIQ-Cog-P increased by 1 point and the ICIQ-Cog by 0.8 points (Table 4). This means a worsening of the nursing burden by 6.7% and of the patient’s quality of life by 2.7%. The correlations were statistically significant (*p* < 0.05).

### 3.3. Correlation between the ICIQ-Cog-P and the ICIQ-Cog-C

The ICIQ-Cog-P highly positively correlated with the ICIQ-Cog-C. The Pearson correlation coefficient was r = 0.62 (95% CI 0.43; 0.76). In the linear regression model, a 1-point increase in ICIQ-Cog-P led to a 0.27-point increase in ICIQ-Cog-C (Table 4). The correlations were statistically significant.

## 4. Discussion

In an aging society with a high number of cognitively impaired persons in nursing homes, incontinence and its care are becoming more and more important. Incontinence has a negative impact on the quality of life of the affected persons and their caregivers [3]. Furthermore, the socioeconomic burden will increase due to the increasing costs of incontinence care and treatments for its complications [13]. Up to now, there has existed no tool for assessing the disease-specific impact on those affected, as existing questionnaires are not tailored to the specific living situations of cognitively impaired people in care facilities [17]. Similarly, there has been no tool for recording the burden caused by incontinence on professional caregivers. Therefore, we aimed to investigate the correlations and effects between the severity of incontinence and the quality of life of those affected, and the incontinence-related burden on their caregivers, using the newly developed International Consultation on Incontinence Questionnaire Cognitively Impaired Elderly (ICIQ-Cog) questionnaire.

As an indicator of incontinence severity, we compared the types of incontinence devices used, assuming that larger pads are used for more severe incontinence. However, we were not able to identify any increased burden on those affected and their care in the ICIQ-Cog according to the type of incontinence products. In terms of the percentages, most persons with double incontinence used all-around pants. The lowest number of persons with double incontinence used small pads. The correct choice of incontinence product for the severity and type of incontinence could explain why no differences in the burden could be measured here [21,22]. Another reason why no differences could be confirmed may be related to the quality of the incontinence material used. The functionality is different between superabsorbent pads and fluff pulp products [23]. We did not capture these product quality differences. Similarly, the correct time of changing the device can impact the care effort and thus the burden placed on the persons involved. If the change takes place too late, the extent of care required can be multiplied due to the need to change bed linen and/or clothing [24]. Although our study asked whether the number of pads used per day was recorded and 83.8% answered this question with yes, the ICIQ-Cog did not ask for the precise number. However, the number of pads used per 24 h alone, without considering the pad weight, would also not yield a conclusion about the filling state of the changed pads, because the number of pads used per 24 h does not correlate with the amount of involuntary urine loss [25]. The documentation of fluid intake, micturition volume, or pad weight in a BD was performed in only 4 of the 60 cases in our study. This also led to the high quantity of missing data related to the questions about incontinence episodes, as these questions were linked to a BD in our questionnaire. Nevertheless, some nurses answered the questions and documented the episodes without keeping a BD. In the four cases in which a BD was kept, it was part of the officially used documentation software of the nursing facility. The development of an electronic BD in addition to the ICIQ-Cog would be very useful in order to better verify medical and nursing interventions. Such an electronic BD should be able to record the amount of fluid intake and the weight/volume of excreted urine/stool and transfer it to an app automatically. This would reduce the documentation-related effort required on the part of nurses and thus increase the acceptance of the BD [26]. The tool could also be applied to the diagnostics and therapeutic control of multiple other lower urinary tract disorders.

As a further indicator of the severity of incontinence, we examined the type of incontinence. We analyzed whether patients or their caregivers experienced a higher burden associated with additional fecal incontinence compared to patients with only urinary incontinence. We were unable to confirm this based on our data. In our setting, no different disease-specific burden was demonstrated in the ICIQ-Cog due to additional fecal incontinence. However, in the home care setting, an increased burden seems to result from fecal incontinence, which has been reported as a reason for hospitalization and nursing home stays [27]. In contrast, Finne-Soveri et al. showed no increased subjective burden among family caregivers despite an increased care time burden due to fecal incontinence in the home care setting for professional caregivers and family members [11]. The subjective burden on the informal caregivers and family members was significantly influenced by impaired cognition and physical impairment. The subjective burden on professional caregivers was not examined in that study [11]. Overall, studies assessing the caregiving situation of doubly incontinent individuals with cognitive impairment are rare [11,28]. Those investigating the subjective burden of double or fecal incontinence on professional caregivers do not exist, to our knowledge.

According to our results, the most important aspect of the subjective burden for professional caregivers and for the affected persons does not seem to be the type of incontinence but the frequency of the incontinence episodes themselves, including the resulting care effort. Particularly, regarding nocturnal incontinence episodes and the proportion of incontinence-related care out of total care, we demonstrated significant effects on the ICIQ-Cog-P and -C. We were able to show the extent to which the burden on the affected persons and their caregivers can be anticipated to change when the incontinence episodes at night or the proportion of incontinence care out of total care increases. Thus, a reduction in incontinence episodes and a reduction in incontinence-related nursing workload could contribute to a reduction in the workload of all involved. For example, a significant 35% reduction in incontinence episodes and thus a 42% reduction in caregiver workload was achieved when the rectum was completely emptied, regardless of the type of drug treatment used or laxative measures [29]. Schnelle et al. demonstrated that improved stool frequency also reduced incontinence [30]. A prospective study on the effect of prompted voiding on urinary incontinence demonstrated an increase in the number of continent bowel movements and a percentage increase in continent bowel movements [31]. As early as 2002, Schnelle et al. investigated the effects and burdens of personalized incontinence care and physical exercise among nursing home residents in a randomized controlled trial [32]. Despite the long-published studies of Schnelle et al. and Chassagne et al. on the reduction in incontinence [29,30,32], neither a validated questionnaire on the burden of urinary or double incontinence on professional caregivers nor an instrument for third-party assessment in people with cognitive impairment has been developed.

Currently, the shortage of nursing staff is increasing [33]. Additionally, the expected increase in the need for nursing home care for elderly people was up to 50% as early as 2002 [32]. Nevertheless, we are still unable to prove whether and to what extent therapeutic and nursing measures influence incontinence care and its burden. Research must be carried out on more efficient care measures in order to maintain satisfactory living situations for all concerned persons in the future. The ICIQ-Cog, the validation process for which is currently continuing in English and German, could serve as a tool to document the changes in quality of life and burdens for both groups of persons and to develop adapted strategies for more efficient care of incontinence.

## 5. Conclusions

Incontinence episodes per night and the proportion of incontinence care out of total care showed significant correlations with the patient- and caregiver-related scores on the ICIQ-Cog. Both items had negative effects on patient quality of life and caregiver burden. According to our results, improving nocturnal incontinence and reducing the proportion of incontinence care out of total care could be an approach to reduce the incontinence-specific suffering of affected patients and their professional caregivers. However, a separate study with a specific design is needed for validation. In times of increasing shortages of nursing staff and the growing nursing-related requirements of the population, there is a need for further research on more efficient incontinence care measures. The ICIQ-Cog could help to verify medical and nursing interventions.

## 6. Limitations

One limitation was the gender imbalance in our study population. The proportion of women in our study was 83.3% and was higher than the average in German nursing homes of 72% [34]. A reason for the elevated proportion of women may be that the inclusion criteria for urinary incontinence, which has a higher prevalence among women [35], biased the gender composition in this open observational study. Another limitation is the high number of missing observations for the frequency of nocturnal episodes and incontinence episodes for 24 h caused by the question character of the ICIQ-Cog. This finding will be reflected in subsequent versions of the questionnaire. Although the achieved sample size of this study is rather small, the post hoc power analysis for the primary outcome (correlation between the number of nocturnal incontinence episodes and the sum score of the ICIQ-Cog-P and -C) revealed acceptable power of 0.88 and 0.76, respectively (significance level 0.05, *n* = 16, and correlation coefficients of 0.69 and 0.62, respectively). Due to the small number of cases, additional confounding factors were not included in the linear regression model for correction.

## Figures and Tables

**Table 1:** Patients’ and caregivers’ characteristics (MMSE—Mini-Mental State Examination, GDS—Global Deterioration Scale).

Patients	Mean (SD)	Median (25–75%)	Min; Max	Missing% (*n*)
Age of patient in years	86.67 (7.39)	88.5 (82.5; 91.0)	62; 100	0 (0/60)
MMSE	11.77 (9.54)	11.0 (0.5; 19.75)	0; 29	0 (0/60)
GDS	4.77 (1.72)	5.0 (3.0; 6.0)	1; 7	5.3 (3/60)
School education in years	10.84 (2.55)	11.0 (8.0; 13.0)	8; 17	43.3 (26/60)
Incontinence duration in years	2.65 (2.63)	2.0 (0.0; 4.0)	0; 12	8.3 (5/60)
Duration of stay in months	41.85 (36.05)	39.0 (12.25; 52.75)	0; 165	0 (0/60)
Number of concomitant diagnoses	10.23 (6.23)	9.0 (5.25; 14.0)	1; 31	0 (0/60)
Frequency of incontinence episodes/night	2.19 (1.34)	2.0 (1.25; 3.75)	0; 4	73.3 (44/60)
Frequency of incontinence episodes/24 h	3.54 (0.88)	4.0 (3.25; 4.00)	1; 4	60.0 (36/60)
Proportion of incontinence care/total care	41.44 (20.36)	45.0 (25.0; 60.0)	0; 80	1.7 (1/60)
Caregivers	Mean (SD)	Median (25–75%)	Min; Max	Missing% (*n*)
Age of caregiver in years	43.83 (7.09)	48.0 (38.0; 48.0)	31; 60	0 (0/60)
Work experience in years	15.28 (6.83)	13.0 (10.0; 13.0)	1; 30	0 (0/60)
Amount of care in hours/week	29.84 (15.75)	38.5 (8.0; 38.5)	3; 64	1.7 (1/60)

**Table 2:** Description of the results for the ICIQ-Cog-P and -C.

	Mean (SD)	Median (25–75%)	Min; Max	Missing% (*n*)
Summed score ICIQ-Cog P	23.22 (7.85)	23 (17.0; 28.0)	12; 48	0 (0/60)
Summed score ICIQ-Cog-C	10.78 (3.43)	10.5 (7.25; 13.0)	5; 16	0 (0/60)

**Table 3:** Summary of the correlations between the type of incontinence and the types of incontinence devices used with ICIQ-Cog-P and -C.

	Type of Incontinence	*n*	Min; Max	Mean (SD)	Median (25–75%)	Two-Sample *t*-Test (95% CI) *p*-Value
ICIQ-Cog-P	Urinary	25	13; 43	23.52 (7.33)	24 (18; 28)	0.52 (−3.55; 4.59) *p* = 0.799
	Double	35	12; 48	23 (8.3)	21 (17; 27.5)	
ICIQ-Cog-C	Urinary	25	5; 16	9.84 (3.2)	10 (7; 12)	−1.62 (−3.36; 0.12) *p* = 0.068
	Double	35	7; 16	11.46 (3.48)	12 (8; 15)	
	Type of incontinence device					
ICIQ-Cog-P	Others	55	12; 48	23.45 (8.0)	23 (17; 28)	2.0 (−4; 10) *p* = 0.547
	Pants	5	16; 31	20.6 (5.94)	19 (18; 19)	
ICIQ-Cog-C	Others	55	5; 16	10.8 (3.56)	10 (7; 13.5)	0 (−3; 4) *p* = 0.925
	Pants	5	8; 12	10.6 (1.67)	11 (10; 12)	

**Table 4:** Summary of the correlations between incontinence episodes per night/24 h and proportion of incontinence care out of total care with ICIQ-Cog P and -C, and the correlation between ICIQ-Cog-P and ICIQ-Cog-C.

	ICIQ-Cog	*n*	Pearson Coefficient (r) (95% CI)	Impact of Incontinence Episodes/Care on ICIQ by Increase of 1 Unit (95% CI) *p*-Value
Incontinence episodes/night (times)	ICIQ-Cog-P	16/60	0.69 (0.3; 0.88)	4.78 (1.91; 7.65) *p* = 0.003
	ICIQ-Cog-C	16/60	0.62 (0.19; 0.86)	1.22 (0.35; 2.1) *p* = 0.01
Incontinence episodes/24 h (times)	ICIQ-Cog-P	24/60	−0.07 (−0.46; 0.34)	-
	ICIQ-Cog-C	24/60	0.16 (−0.26; 0.53)	-
Proportion of incontinence care/total care (%)	ICIQ-Cog-P	59/60	0.27 (0.01; 0.49)	0.103 (0.004; 0.20) *p* = 0.042
	ICIQ-Cog-C	59/60	0.46 (0.23; 0.64)	0.078 (0.04; 0.12) *p* < 0.001
ICIQ-Cog-P	ICIQ-Cog-C	60/60	0.62 (0.43; 0.76)	0.27 (0.18; 0.36) *p* < 0.001

## Data Availability

The data presented in this study are available on request from the corresponding author. The data are not publicly available due to privacy.
